# Supplementary material for: Reaction moments matter when designing lower-extremity robots for tripping recovery
Source: PLoS One. 2023 Feb 21;18(2):e0280158. doi: 10.1371/journal.pone.0280158 (PMC9942996; doi:10.1371/journal.pone.0280158)
Supplement: Attachment — Submitted filename: Response to Reviewers.pdf [file pone.0280158.s007.pdf]

Date October 8, 2022
Contact Patricia M. Baines
Phone +31 646117155
E-mail p.m.baines@tudelft.nl
Subject Revision article

Faculty of Mechanical, Maritime and Materials Engineering,
Department of BioMechanical Engineering

Mekelweg 2 (Building 34)
2628 CD Delft

PO Box
Postbus 5
2600 AA Delft

PLOS ONE
Editorial Manager

Dear Dr. Tischer,

Thank you for your careful consideration of our manuscript. We found the suggestions of the editor and reviewers to be helpful and highly insightful and would like to thank you for the opportunity to submit a revised version of our manuscript.

Revisions in the manuscript are highlighted in blue text in 'Revised Manuscript with Track Changes', and below, you will find our responses to each comment in detail.

Journal Requirements:

Comment 1: Please ensure that your manuscript meets PLOS ONE's style requirements, including those for file naming.
Response: We have revisited the style requirements and ensured correct file naming. Based on the provided links we made some minor changes on the title page and we have used the LaTeX PLOS ONE template found at
https://journals.plos.org/plosone/s/latex

Comment 2: Thank you for stating the following in the Acknowledgments Section of your manuscript: *"This research work was supported by the Netherlands Organisation for Scientific Research (NWO) Innovational Research Incentives Scheme Vidi grant 14865."* However, funding information should not appear in the Acknowledgments section or other areas of your manuscript. We will only publish funding information present in the Funding Statement section of the online submission form. Please remove any funding-related text from the manuscript and let us know how you would like to update your Funding Statement. Currently, your Funding Statement reads as follows: *"This research work (HV,SJ) was supported by the Netherlands Organisation for Scientific Research (NWO) Innovational Research Incentives Scheme Vidi grant 14865. The funders had no role in study design, data collection and analysis, decision to publish, or preparation of the manuscript."* Please include your amended statements within your cover letter; we will change the online submission form on your behalf.

Response: We agree with the information in the funding statement. Thank you for providing it. We have removed funding information from the Acknowledgements section.

Comment 3: We note that you have stated that you will provide repository information for your data at acceptance. Should your manuscript be accepted for publication, we will hold it until you provide the relevant accession numbers or DOIs necessary to access your data. If you wish to make changes to your Data Availability statement, please describe these changes in your cover letter and we will update your Data Availability statement to reflect the information you provide.
Response: Thank you for the information. A repository containing all the documented simulation input files, the data extracted from simulation output, and the scripts to extract and visualize the data can be accessed via a private link at 4TUD data repository (doi:10.4121/21197149 will be activated once the repository is made public). These files are available to use under the Apache 2.0 license.

Response to Reviewer 1:

Comment 1: This is the study that simulated the recovery process of the gait after tripping. The simulator applies recovery torque to the trunk, thigh, and shank. The effect of assisted links and assist torque on the performance of recovery motion, such as MoS and the number of recovery steps. Such parametric study is important to investigate the sensitiveness and mechanism of recovery motion.
Response: Thank you for the recognition of our efforts. This was indeed our intent.

Comment 2: The application of free moment to the wearable device does not seems feasible. Although the reaction wheel, control moment gyro, and thruster are refereed, they were not used to assist specific body links such as thigh. Furthermore, as the principle of these mechanisms, sufficient mass is required to generate torque. It is a big disadvantage of the wearable device. In addition, the inertia of rotating body of these mechanisms will disturb the gait motion. I think the other actuator is required to apply free moment.
Response: The reviewer's concerns over the technical feasibility of external-reaction wearable actuators are certainly valid. The section 'Discussion: Practical application: External-reaction wearable actuators' has been rewritten to more explicitly address these points:

> ... However, the principle of operation also introduces several limitations. The presence of a passive mass for energy storage, and sometimes multiple motors (control moment gyroscopes), can entail that inertial-manipulating actuators are often larger and heavier than alternatives with similar power output. While the detrimental effects of added mass on gait is similar for all wearable devices, actuators with rotational energy storage (e.g., reaction wheels and control moment gyroscopes) also have the potential to disturb gait through unintended gyroscopic moments induced through rotational movement of the actuated body segment – however, these induced moments can be partly or wholly cancelled internally via mechanical constraints (Brown et al. 2008) or control (Valk et al. 2018) or even be exploited to produce intentional changes in gait patterns (Jabeen et al. 2021). Finally, although all actuator types are limited in their maximum output moment and power,

> inertia-manipulating actuators also have impulse constraints that limit the duration of moment application and prevent them from providing continuous support in static directions.
>
> Given the relatively recent appearance of these technologies in wearable applications, there are few empirical analyses and it is yet unclear which actuation architecture is best (or even feasible) for practical implementation. For example, most empirical studies involving control moment gyroscopes have thus far sought to influence balance through control of the motion of the CoM (Chiu et al. 2014, Lemus et al. 2020, Romtrairat et al. 2020), whereas control of the lower extremities has been investigated only in simulations (Jabeen et al. 2019). However, parallel research has explored the uses of these actuators on the limbs for other purposes, such as tremor-suppression (GyroGlove by GyroGear; London, UK), upper-extremity prostheses (Jarc et al. 2006, Muller et al. 2017), and emulation of virtual environments (Duda et al. 2015). Ongoing research continues to investigate the extent to which optimisation of mechanical designs can mitigate technical limitations and exploit the proposed advantages (Meijneke et al. 2021). While much development and validation of these emerging technologies remains, they may soon fulfill a complementary roll in the technological landscape and offer versatile solutions for facilitating limb movement and balance.

At the Delft Biorobotics Laboratory at TU Delft, extensive research has been performed to optimize the design of wearable gyroscopic actuators to address these technical challenges.

Comment 3: The evaluation index of recovery is not elaborate. The number of recovery steps counts the steps before fall. Thus, the number became 22 for all the trials that did not fall. It is difficult to evaluate the timing of recovery. It is required to determine the physical index of "balance recovery" and evaluate the difference of the timing of recovery among trials.
Response: Indeed, our selection of outcome measures needs further elaboration. The binary presence/lack of a fall and the number of steps before a fall are certainly not comprehensive measures of balance performance. To make our approach clear, we have added substantial text to explain the reasoning for the primary outcomes and bring greater emphasis to the role of the secondary outcome measures for interpreting responses in terms of kinematic and kinetic movement characteristics.
In the case, gait is sustained (i.e., no fall occurs), there are several other measures of disturbance-rejection available, such as the number of steps until periodicity, the Gait Sensitivity Norm, and Lyapunov exponents of a Poincaré section of the orbital dynamics, among others. However, when the disturbance is not successfully rejected (i.e., the intervention does not successfully prevent a fall), these measures have limited use. Instead, it is relevant to consider the degree to which a fall is resisted. This is the case in the present study since we wish to compare interventions for preventing falls.
To clarify which simulations successfully avoided falling, we have replaced the upper ceiling of 22 steps with the designation 'Full Recovery' (FR). This is because the simulation horizon is arbitrary, since any simulated perturbation followed by more than 5 steps would always converge back to cyclical gait.
The section 'Methods: Performance measures' has been modified to reflect these clarifications:

> The primary criterion for assessing the efficacy of each intervention was whether or not the intervention prevented a fall following a tripping event.

> Although this is not a comprehensive metric of balance proficiency, it is a practical and unambiguous measure of *functional* performance with a high face validity for fall avoidance. Simulations in which a fall was successfully avoided were designated 'Full Recovery' (FR).
>
> When a fall is not successfully avoided, the characteristics of the response are still relevant for analysis. Even if external assistance does not prevent a fall in simulation, it can mitigate the effects of the perturbation and prolong gait so that additional, real-world action could potentially be taken to prevent injury or sustain gait – for example, affording the wearer more time to react and execute their own postural response, complementary to the external assistance. Therefore, the number of steps between the tripping event and the falling event was recorded as a secondary outcome. Steps were counted with respect to the first heel-strike after perturbation until either a body segment other than the foot contacted the ground or the heel of the swing foot was placed posteriorly to the toe of the stance foot.

and the number of steps for 'Full Recovery' is clarified in the section 'Methods: Assistance':

> If the model continued to walk for at least ten steps after obstacle contact, it was considered to have fully recovered – preliminary simulations showed that falls always occurred within five steps of perturbation, similar to the number of steps required by healthy young adults to normalize gait following tripping (Forner Cordero et al. 2003).

We have also added further description of our secondary outcome measures, including reference to prior studies that have made use of them. In addition to the existing text explaining the role of the MoS to describe and interpret the physical response to tripping and assistance, we have added the following text to describe our use of joint kinematics:

> The kinematic responses in the first step following perturbation were also analyzed. Kinematic features such as the trunk forward lean angle and joint kinematics are widely used to differentiate between fallers and non-fallers (van Dieën et al. 2005, Pavol et al. 2001, Grabiner et al. 1993). Kinematic responses are reported for a gait cycle starting at toe-off of the perturbed (TO-PL) limb before perturbation and ending at toe-off of the same limb after perturbation (Fig 1a). The trunk forward lean angle is measured from the vertical axis and is reported as a positive angle when the body leans forward. The hip flexion angle and knee flexion angle are presented along with the lengths of the first steps of the perturbed (swing) limb (SL_P) and the trailing (non-perturbed) limb (SL_T).

While a single, universal measure of balance performance unfortunately remains elusive, we believe this combination of functional measures and kinematic/kinetic measures sufficiently describes the principal features differentiating the different modes of lower-extremity assistance.

Comment 4: The validity of the gait algorithm is unclear. It is mentioned that the non-assisted gait cannot recover from the smallest perturbation. Perhaps, it is because the gait algorithm was not developed for simulating the recovery from tripping. However,

of cause the healthy adult usually can recover from small perturbation without assistance. The analysis of recovery motion should consider the human's general ability to recover from tripping.

Response: The gait algorithm has indeed been previously validated in a number of empirical studies, including with tripping perturbations. The following text and references have been added to the section 'Methods: Simulation tool':

> ... The controller used to generate walking patterns for this study has been widely validated in different studies to analyse aspects of impaired gait, such as the effects of aging on balance control (Song et al. 2018, Reimann et al. 2020) and muscle activity (Ping et al. 2021). The model has also been validated in response to mechanical disturbances, such as tripping by constraint of the swing leg and slipping of the stance leg, yielding response trends similar to experimental data for the majority of muscles (Song et al. 2017). ...

We clarify that the magnitudes of the perturbations were specifically set to induce falling in both 'Method: Simulation tool':

> Although the generated gait patterns were robustly stable to slight deviations, the walking controller was not optimized to recover the balance from large perturbations, and the model would fall if no intervention was made. This represents a scenario in which a human would need support to avoid falling.

and 'Methods: Perturbations':

> ... To explore the efficacy of different interventions for preventing falls after tripping, the perturbations were designed such that the model could not sustain gait in the absence of external assistance.

However, a model is indeed an idealization of reality and may not perfectly the represent the response to external moments applied to the lower extremities. This is stated in the section 'Discussion: Study limitations and future work':

> The SCONE planar simulation model and underlying reflex model has been previously validated against human data for unperturbed walking and certain types of balance disturbances (Waterval et al. 2021). However, it is unclear the extent to which dissimilarities in the experimental protocols (e.g., the mode, magnitude, and timing of balance disturbance) and different choice of outcome measures would affect the model validity. For example, trunk kinematic responses are critical for distinguishing fallers from non-fallers during tripping responses (Pavol et al. 2001), yet here the upper body was modelled as a single segment, in which the pelvis, vertebrae, head, and arms were rigidly fused together. Qualitative comparison of our simulation results (unassisted condition) with empirical studies show a similar trend between peak trunk lean angle and the magnitude of the tripping perturbation (Grabiner et al. 1993, Graviner et al. 1996) and similar characteristics of the knee and hip joint responses following tripping (Grabiner et al. 1993). Nevertheless, a quantitative comparison is lacking and there is currently no data to validate the neuromechanical response to external moments applied at different

locations on the body. It is thus far unclear whether moments produced by wearable actuators could, under any circumstances, initiate additional, unmodelled reflexes that might change the response characteristics. In addition, a JM hip actuator would conventionally attach to the thigh and pelvis, but, because the pelvis and trunk are fused in the simulation model, the simulation may falsely exaggerate trunk lean when assistive moments are applied.

Comment 5: The validity of gait simulator should be discussed. The qualitative and quantitative investigation should be done by comparing simulation output with the motion observed in the actual tripping experiment reported previously.
Response: Both the reflex-based gait model and the SCONE simulation environment have been validated in several studies. In addition to clarifying the validity and limitations of the gait model (please see the response and quotations of the previous comment), we have clarified the validity of the SCONE implementation in 'Methods: Simulation tool':

> ... The SCONE implementation of this model has also been validated against normative data (Waterval et al. 2021). ...

A full list of publications using SCONE is available at
https://scone.software/doku.php?id=publications .
In the section 'Discussion: Study limitations and future work' we also compare the simulated kinematic responses to tripping with empirical studies – please see the last quote in the response to the previous comment.

Comment 6: Generally, tripping experiments use vertical plate. Why was the spherical obstacle used?
Response: This was one of the limitations of the SCONE simulation environment. At the time of this study design, it was not possible to encode a step-like object to simulating tripping, hence a spherical object was used instead. This limitation is now mentioned in the section 'Methods: Perturbation':

> A spherical obstacle of varying diameter (shape constrained by the software at the time of writing) was fixed to the ground surface to obstruct the swing leg and produce tripping events (Fig 2).

Comment 7: The timing to apply assist torque seems unclear. How many gait cycles be the assist torque applied to?
Response: The assistive torque was applied to only one gait cycle – the same cycle in which the model is perturbed but after the foot has cleared the obstacle. The duration of the assistance was fixed but the onset time was varied to maximize the number of steps recovered following perturbation. This has been clarified in the section 'Methods: Assistance':

> We tested 5 different types of assistance in which either a flexion moment on the thigh or an extension moment on the shank was applied to assist forward placement of the foot of the obstructed leg. This assistance was applied unilaterally to the perturbed limb during the swing phase of the obstructed step but after obstacle contact.
>
> ...

> For comparing actuator types and placement, all moments were parametrized as an ideal square wave of magnitude M, start time within the perturbed swing phase t_s, and sustained duration ΔT (Fig. 1b). A range of values of these three variables was simulated to determine the best parameters for each use case. Considering the results obtained from a previous simulation study (Jabeen et al. 2019) the duration of the assistance was set to 200 ms, peak moment limits [0,20] N m with intervals of 2 N m, and start time is with respect to foot and obstacle clearance. The assistance was initiated after the foot cleared the obstacle, with start times in [0,210] ms (30 ms intervals).

Comment 8: The method to tune control parameters explained in Fig. 3 is ambiguous. What's the objective function of this optimization?
Response: The objective function was not a continuous function, but rather a hierarchical piece-wise set of rules. To select the control parameters, this rule set was evaluated using an exhaustive grid search over a pre-defined discrete parameter space. We have added clarification how we tuned the control parameters to the text of section 'Methods: Assistance':

> To compare the best-case realization of each actuator type and placement, an exhaustive grid search was performed to determine the control parameters (assistance magnitude and start time) that maximized the number of steps completed following the tripping event. The perturbation was applied halfway through a walking simulation of approximately 30 s, in which the latter 15 s for the model to get back in cyclic motion. If the model continued to walk for at least ten steps after obstacle contact, it was considered to have fully recovered – preliminary simulations showed that falls always occurred within five steps of perturbation, similar to the number of steps required by healthy young adults to normalize gait following tripping (Forner Cordero et al. 2003). In the event of multiple candidates for a full recovery, the parameter set was selected that first minimized the assistance magnitude and then minimized assistance start time (Fig 3). In the case of partial or failed recovery (model walked only a few steps and fell), the assistance parameter set was selected with maximum anterior margin of stability (MoS) (Hof et al. 2005) of the recovery step. Fig 2 shows the gait cycle and MoS definitions used for the analysis and the gait events in terms of perturbed and recovery limb.

and modified Fig. 3:

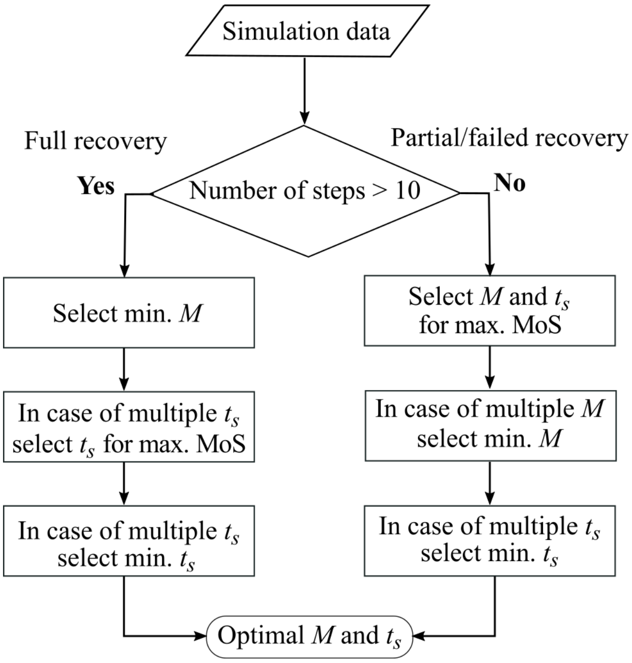

Fig 3. Workflow for selection of best-case assistance parameters. Selection procedure of assistance parameters magnitude M and start time t_s based on the outcome measures number of steps (n) and margin of stability (MoS) and the values of the assistance parameters.

Response to Reviewer 2:

Comment 1: This is an excellent paper and will make a significant contribution to the growing field of lower-extremity robotic aids.
Response: Thank you for your kind words.

Comment 2: The context for the internal and external moments is unclear until pg 3 (Pg 9 of PDF) when the examples are given to the different types of gait assistive technologies (wearable devices verses stationary devices). It may aid the reader to have this context presented earlier in the Introduction.
Response: We have reduced the amount of background information presented before introducing robotic orthoses and different definitions of reaction moments. The first mention of these now appear in the second paragraph.

Comment 3: Pg 8 of PDF, line 23: Perhaps, but falls in seniors are more precisely linked to transitions to (chair transfers) or from gait (turns, etc.). See work by Stephen Robinovitch at SFU.
Response: In community-dwelling adults, gait is cited as the activity with the largest risk of falling (Blake et al. 1988, van Dieën et al. 2005). However, elderly persons in long-term care, such as in the studies of Robinovitch, are generally less mobile and tend to be confined to indoor spaces. The text originally referred to has been removed, and Paragraph 1 has been clarified to emphasise that we specifically focus on community-dwelling persons with gait impairments. Paragraph 1 now reads as follows:

> ... Amongst community-dwelling individuals, more than 53% of falls occur due to tripping while walking (Blake et al. 1988, van Dieën et al. 2005) ...

Comment 4: Pg 17 of PDF, line 352: Interesting finding. Some recent studies of the Keeogo (BTemia) exoskeleton which is of the $JM_{Sk\text{-}Th}$ type would appear to agree with this - both healthy individuals and patients do not walk faster when wearing the device.
Response: Thank you for referring us to this interesting finding. We have added the following text to the paragraph in question:

> ⟨ Reaction moments on the thigh initiate early foot contact and produce a shorter step length ⟩... This may also contribute to an empirical observation that a powered knee orthosis with joint actuators significantly decreases gait speed in both healthy and impaired users compared to when the device is not worn (although it is unclear to what extent this may be due to the added inertia or impedance of the device) (McGibbon et al. 2018).

Comment 5: Pg 18 of PDF, line 365: Wouldn't this be a knee exoskeleton?
Response: Thank you for bringing this to our attention. This is indeed a typographical error. The authors intended to refer to a hip exoskeleton, properly denoted as $JM_{Th\text{-}Tk}$ instead of $JM_{Sk\text{-}Th}$. This is fixed in the manuscript:

> ... however when the reaction moment is applied on the trunk, as with a traditional hip exoskeleton ($JM_{Th\text{-}Tk}$), ...

If you have any questions or if anything is missing from our submission, please do not hesitate to contact me.

With kind regards,

Patricia M. Baines
