# Supplementary material for: Reaction moments matter when designing lower-extremity robots for tripping recovery
Source: PLoS One. 2023 Feb 21;18(2):e0280158. doi: 10.1371/journal.pone.0280158 (PMC9942996; doi:10.1371/journal.pone.0280158)
Supplement: Attachment — Submitted filename: Response to reviewers.pdf [file pone.0280158.s008.pdf]

Date December 17, 2022
Contact Patricia M. Baines
Phone +31 646117155
E-mail p.m.baines@tudelft.nl
Subject Article 2nd revision

Faculty of Mechanical, Maritime and Materials Engineering,
Department of BioMechanical Engineering

Mekelweg 2 (Building 34)
2628 CD Delft

PO Box
Postbus 5
2600 AA Delft

PLOS ONE
Editorial Manager

Dear Dr. Shaheen,

Thank you for your continued consideration of our manuscript. We would like to thank you for the opportunity to submit a revised version of our manuscript and both you and the reviewers for your careful scrutiny. We find the comments to be constructive towards finalising this manuscript.

Revisions in the manuscript are highlighted in blue text in ‘Revised Manuscript with Track Changes’, and below, where you will find detailed responses to each comment and reprints of the relevant excerpts.

Journal Requirements:

Comment 1: Please review your reference list to ensure that it is complete and correct. If you have cited papers that have been retracted, please include the rationale for doing so in the manuscript text, or remove these references and replace them with relevant current references. Any changes to the reference list should be mentioned in the rebuttal letter that accompanies your revised manuscript. If you need to cite a retracted article, indicate the article's retracted status in the References list and also include a citation and full reference for the retraction notice.

Response: The reference list has been checked for completeness and minor inconsistencies have been rectified. To the best of our knowledge, none of the articles in our references list have been retracted and all articles are currently accessible via the respective publisher's website. Changes to the References list are described in the response to Reviewer 2 below.

Additional Editor Comments:

Comment 1: Please make this final review of the article in relation to the comments made by the reviewer regarding the accuracy of representing previous studies.

Response: We have addressed these concerns. Please see the response to Reviewer 2 below.

Response to Reviewer 2:

Comment 1: Although the authors adequately addressed all the comments from this reviewer, it would behoove the authors to become more familiar with the paper they cited (McGibbon et al. 2018) which is implied to include healthy subjects, but as far as I can tell it only includes people with multiple sclerosis. Also, the claim being made about "significantly reduced gait speed" is technically incorrect (the outcomes measures are functional tests: 6mwt, TUG test, and stair test). To be clear about my original comments: As far as this reviewer knows, no studies have shown an increase in walking speed whilst using this type of exoskeleton, but decrements in walking speed appear to vary depending on the population being studied.

Response: Thank you for your careful scrutiny. Indeed, we erroneously cited McGibbon et al. 2018 as including healthy subjects, although the article addressed only impaired participants, and we misleadingly implied that the scores of functional tests such as the 6-Minute Walk Test (6MWT) – or even the Timed Up-and-Go (TUG) test and Timed Stair Test (TST) – accurately and unambiguously represent gait speed alone.

The TUG and TST, although measures of locomotive speed between two points, are not strictly measures of gait speed because they include movements other than gait (TUG) or require the subject to traverse complex terrain instead of level ground (TST). The 6MWT is, superficially, a measure of mean gait speed over a 6-minute interval – McGibbon et al. 2018 even convert gait speed from an earlier study into a hypothetical 6MWT score; however, the recorded score (the distance walked) is also influenced by the stated objective ('walk as far as you can', not 'walk as fast as you can') and poorly ambulatory subjects may be liable to change gait speed, pause, or stop walking before the entirety of the 6 min has elapsed. Therefore, the 6MWT may be interpreted as a combined measure of ambulatory endurance and self-selected sustained gait speed, rather than gait speed alone. The 10 m Walk Test (10MWT) is a more conventional clinical measure of comfortable and fast voluntary gait speed that puts less emphasis on endurance; however, this test was not reported in the articles that we examined.

Although we have not exhaustively searched the literature for studies empirically evaluating the effects of active knee orthoses on gait performance, a number of articles by McGibbon et al. document the impact of the Keeogo exoskeleton, both with and without training. Their studies involving impaired populations, however, report only functional outcomes that are only speculatively related to gait speed, so we have chosen not to cite them in this context. We have changed the existing citation to refer to a study involving unimpaired individuals that does explicitly measure gait speed, McGibbon et al. 2017. We have further clarified our assumptions and reasoning for linking our simulations with these published results. The text in question (pp. 12-13 of the manuscript without markup) has been changed to the following:

> Both actuators with an action moment on the shank (FM_{Sk} and $JM_{Sk\text{-}Th}$) extend the knee joint (Fig 6) with the intention of bringing the foot forward with respect to the knee and increasing anterior foot placement; however when a reaction moment is simultaneously applied to the thigh ($JM_{Sk\text{-}Th}$), hip flexion is inhibited during the mid-swing phase and/or hip extension oc-

curs prematurely during the late-swing, depending on when the assistance is initiated. This decreased flexion or increased extension lowers the knee with respect to the pelvis and, consequently, lowers also the foot and causes early ground contact. During the swing phase, the pelvis forward velocity is not substantially affected by the assistance, so early swing termination decreases step length (S1 Table) and creates a smaller anterior moment arm between the CoP and CoM, lessening the centroidal retarding moment needed to counteract the effect of the perturbation. This is evidenced by a decreased anterior MoS and number of successful steps when compared to FM_{Sk} in several of the perturbation conditions (Table 2 and Figs 4 and 5) and the finding that $JM_{Sk\text{-}Th}$ is often most effective when the assistance is too low or late to influence foot placement (Fig 5).

Incidentally, if cadence remains similar, a decreased swing time and step length due to knee joint assistance may also explain the empirical finding that unimpaired users of powered knee orthoses do not walk faster than when not wearing a device, contrary to the expectation that this would augment physical performance (although it is unclear to what extent this may also be due to the added inertia or impedance of the device) (McGibbon et al. 2017).

We hope that this sufficiently addresses your concerns, and we welcome any further feedback that you may have.

If you have any questions or if anything is missing from our submission, please do not hesitate to contact me.

With kind regards,

Patricia M. Baines
